# Differences in toe flexor strength and foot morphology between wheelchair dependent and ambulant older people in long-term care: a cross-sectional study

**DOI:** 10.1186/s13047-021-00458-8

**Published:** 2021-03-12

**Authors:** Mieko Yokozuka, Sei Sato

**Affiliations:** 1 Preparing Section for New Faculty of Medical Science, Fukushima Medical University, 10-6 Sakae-machi, Fukushima City, Fukushima 960-8516 Japan; 2 Department of Hygiene and Preventive Medicine, Fukushima Medical University, 1 Hikariga-oka, Fukushima City, Fukushima 960-1295 Japan

**Keywords:** Toe flexor strength, Foot morphology, Frail older people, Wheelchair, Mobilization

## Abstract

**Background:**

Hallux valgus, lesser toe deformity, and muscle weakness of the toe flexors contribute to falls in older people. This study aimed to examine the differences in toe flexor strength and foot morphology in older people requiring long-term care due to changes in the way they mobilize in everyday life.

**Methods:**

This study included 84 people aged ≥70 years without motor paralysis who underwent rehabilitation. They were divided into those who could mobilize without a wheelchair (walking group, *n* = 54) and those who used a wheelchair to mobilize (wheelchair group, *n* = 30). The presence or absence of diseases was confirmed, and hand grip strength, toe flexor strength, and foot morphology using the foot printer were measured. The presence of diseases, hand grip strength, toe flexor strength, and foot morphology were compared between the two groups. Multiple logistic analysis was performed with wheelchair dependence as the dichotomous outcome variable, and the percentages of each strength measure observed in the wheelchair group to the average hand grip and toe flexor strength measures in the walking group were compared.

**Results:**

No significant between-group difference in foot morphology was found. The factors related to the differences in ways of ambulating in daily life were history of fracture, heart disease, and toe flexor strength. After comparing the muscle strength of the wheelchair group with the mean values of the walking group, we found that the toe flexor strength was significantly lower than the hand grip strength.

**Conclusions:**

Older people who used a wheelchair to mobilize have significantly less toe flexor strength than those who do not despite no significant difference in foot morphology. Use of a wheelchair is associated with a reduction in toe flexor strength.

## Background

Falls occur in 30–60% of older people each year, of which 10–20% could lead to injuries, hospitalizations, and/or death [1]. The reported odds ratios for falls range from 1.67 to 1.95 in community-dwelling older people with foot problems, such as foot pain, hallux valgus, and lesser toe deformities [2]. In a meta-analysis, the prevalence of hallux valgus in people aged ≥65 years was 35.7% [3]. Hallux valgus was found in 28.4% of older Japanese people living in the community [4]. Although many studies have focused on foot morphology of community-dwelling older people, there are only a few reports focused on this issue in older people that require long-term care. Therefore, the foot morphology of frail older people who need long-term nursing care remains unknown.

Regarding the relationship between foot morphology and muscle strength, it has been reported that the presence of toe deformities and reduced toe flexor strength increase the risk of falls in older people [5]. Correlations have been reported between toe flexor strength and the medial longitudinal arch height [6], toe curl activity [7], and hallux valgus angle [8]. The hallux and lesser toe deformities and the reduction of their muscles affect functional ability; therefore, these issues must be prevented. In addition, the plantar flexor strength of the hallux and lesser toes have been associated with balance and functional ability in older adults [9, 10]. Toe flexor strength is correlated with spatiotemporal gait parameters in community-dwelling older people [11]. Moreover, toe flexor strength was found to be positively correlated with the number of steps taken per day (r = 0.424) [12]. Interestingly, many studies have examined the relationship between activity and toe flexor strength.

Therefore, in those who need long-term care and use a wheelchair for some reason, it is possible that the toe flexor strength is reduced because of the reduced opportunity to perform activities in the standing position. Interventions to maintain toe flexor strength and prevent foot deformities may be necessary, even in a person using a wheelchair, to perform everyday activities safely while standing.

In this study, we aimed to investigate toe flexor strength and foot morphology in frail older people requiring long-term care and examine the characteristics of those who use a wheelchair.

## Methods

### Participants

This cross-sectional study included people aged ≥70 years who were admitted to a healthcare facility for older people requiring long-term care (i.e., the facility provides care services to those who require rehabilitation after hospital discharge and assists them to independently handle everyday challenges) or receiving daily rehabilitation service (i.e., those who visited the health service facility to undergo training for maintenance or recovery of their mental or physical functions and to achieve independent daily living). The study was conducted from January 2020 to April 2020. The study exclusion criteria were motor paralysis, prior hand or foot surgery, inability to stand with or without assistance, and inability to understand simple instructions during measurement of muscle strength and foot morphology.

The study was approved by the Ethics Committee of the Fukushima Medical University (approval number 30293). All participants provided written informed consent after receiving a written and verbal explanation of the purpose of the study.

### Evaluation

Medical history data were collected from the medical certificate or patient referral document.

Toe flexor strength was measured using a toe grip dynamometer (T.K.K.3364b, Takei Scientific Instruments Co., Ltd. Niigata, Japan). The hip, knee, and ankle joints were set at 90° in the chair sitting position, and the ankle joint was fixed with a belt at a position where the bar to be measured could be easily gripped by the toes. To measure the toe flexor strength, the participants were instructed to grip the bar with a maximum force, and the force was held for 2–3 s. The measurement was repeated three times on each side with the bar gripped firmly by the toes. The hand grip strength was measured to compare the proportion of reduction in toe flexor strength using a hand grip dynamometer (T.K.K. 5401, Takei Scientific Instruments Co., Ltd. Niigata, Japan). Measurements were taken twice, on each side, in the chair sitting position with both upper limbs dropped to the sides. The reliability of toe flexor strength [13] and hand grip strength [14] measurements has been previously confirmed. The values for toe flexor strength and hand grip strength divided by body weight were used in the analysis to correct the physique. Additionally, the proportion of hand grip strength or toe flexor strength of an individual in the wheelchair group was calculated as follows:

$$ \mathrm{Proportion}\ \mathrm{of}\ \mathrm{individual}\ \mathrm{hand}\ \mathrm{grip}\ \mathrm{strength}\ \left(\mathrm{or}\ \mathrm{toe}\ \mathrm{flexor}\ \mathrm{strength}\right)\ \mathrm{in}\ \mathrm{the}\ \mathrm{wheelchair}\ \mathrm{group}=\mathrm{individual}\ \mathrm{hand}\ \mathrm{grip}\ \mathrm{strength}\ \left(\mathrm{or}\ \mathrm{toe}\ \mathrm{flexor}\ \mathrm{strength}\right)\ \mathrm{in}\ \mathrm{the}\ \mathrm{wheelchair}\ \mathrm{group}/\mathrm{mean}\ \mathrm{hand}\ \mathrm{grip}\ \mathrm{strength}\ \left(\mathrm{or}\ \mathrm{toe}\ \mathrm{flexor}\ \mathrm{strength}\right)\ \mathrm{in}\ \mathrm{the}\ \mathrm{walking}\ \mathrm{group}\ast 100 $$

For the measurement of foot morphology, being mindful of safety, the participant’s weight was evenly applied to both soles of the feet while sitting on a chair, and the foot contour was taken using a foot printer (Bauerfeind, Zeulenroda-Triebes, Germany). The angle formed by the first phalanx and the first metatarsal bone was measured using a foot contour to obtain the hallux valgus angle. Hallux valgus was present when this angle was ≥16° [15]. For the medial longitudinal arch, the height of the navicular tuberosity from the floor was measured with a right-angle ruler and divided by the foot length to calculate the arch height ratio in the chair sitting position. For the measurement of spread foot, the spread ratio was calculated by dividing the width of the foot by its length. Foot width and length were read from the foot contour. The reliability of the measurement of the arch height ratio [16, 17] and the spread ratio [18] has been confirmed.

All measurements were performed by a physical therapist who was in daily contact with the participants and could communicate with them well.

### Statistical analysis

The participants were divided into the wheelchair group (those using a wheelchair to move in their daily lives) and the walking group (those walking without a wheelchair). Toe flexor strength and foot morphology were analyzed on the side with the greater hallux valgus angle when this angle was ≥16° on one or on both sides. When the angle was < 16° on both sides, the side to be analyzed was chosen using the random number method [19]. For the hand grip strength, the maximum value obtained for the left and right sides was used for the analysis. The distributions of participants’ sex, presence or absence of disease, and hallux valgus in at least one foot were compared using Pearson’s chi-square test. Normality was examined by the Shapiro-Wilk test. Age, hand grip strength, toe flexor strength, and foot morphology were compared using the unpaired t-test or Mann-Whitney U test. Multiple logistic regression analysis with the forward stepwise selection (likelihood ratio) method was performed using the means of movement as the dependent variable and participants’ age and sex, diseases with significant between-group differences, toe flexor strength, and foot morphology as the explanatory variables. To avoid multicollinearity, we examined the correlation coefficients of age, toe flexor strength, hallux valgus angle, arch height ratio, and spread ratio. The paired t-test was used to compare the ratio of the hand grip strength and toe flexor strength in the wheelchair group to the corresponding average values in the walking group. All statistical analyses were performed using SPSS for Windows, version 25 (IBM Corp., Armonk, NY, USA). The significance level was set to 5%.

## Results

Eighty-four older individuals (25 males and 59 females), with a mean and standard deviation (SD) age of 86.4 (6.0) years and a mean (SD) body mass index of 20.7 (3.5), participated in the study. Each participant in the wheelchair group had used a wheelchair for at least 1 month. Table 1 shows the participants’ characteristics and measurements. Significant differences in the fracture rate (*p* = 0.028), frequency of heart disease (*p* = 0.005), and toe flexor strength (*p* < 0.001) were found between the two groups. At least one foot had hallux valgus in 50% (*n* = 27) of the walking group and 53.3% (*n* = 16) of the wheelchair group. No significant difference was noted in foot morphology between the two groups.

**Table 1:** Characteristics and measurements of the study participants presented with their mean (SD) and *n (%)*

	Walking group *n* = 54	Wheelchair group *n* = 30	*p*-value
Age, years	85.78 (6.74)	87.40 (4.45)	0.241
Female, *n (%)*	36 (66.7)	23 (76.7)	0.337
Height, cm	150.19 (8.58)	149.08 (7.43)	0.551
Weight, kg	47.41 (10.59)	45.61 (8.70)	0.447
Disease, *n (%)*			
Bone and joint	27 (50.0)	15 (50.0)	1.000
Fracture	19 (35.2)	18 (60.0)	0.028
Lower limb	10 (52.6)	9 (50.0)	
Spine	8 (42.1)	7 (38.9)	
Upper limb	1 (5.3)	2 (11.1)	
Cerebrovascular	15 (27.8)	9 (30.0)	0.829
Heart	7 (13.0)	12 (40.0)	0.005
Dementia	26 (48.1)	10 (33.3)	0.189
Mental illness	3 (5.6)	2 (6.7)	0.837
Hand grip strength, kg/BW*100	33.54 (9.38)	24.90 (7.52)	< 0.001
Toe flexor strength, kg/BW*100	10.01 (4.26)	5.97 (3.15)	< 0.001
Foot morphology			
Hallux valgus, *n (%)*	27 (50.0)	16 (53.3)	0.770
Hallux valgus angle, °	16.61 (11.17)	19.30 (10.46)	0.193
Arch high ratio, mm/mm*100	22.06 (3.10)	21.43 (3.08)	0.375
Spread ratio, mm/mm*100	41.06 (3.04)	42.10 (2.36)	0.135

Age, height, weight, hand grip strength, toe flexor strength, hallux valgus angle, arch height ratio, and spread ratio are expressed as means (standard deviation)

The number and percentage of female participants, specific disease, and hallux valgus in each group are expressed as *n (%)*

Hand grip strength could not be measured in one participant in the walking group

*BW* Body weight, *SD* Standard deviation

Table 2 shows the results of the multiple logistic analysis using the way of ambulating as the dependent variable, with history of fracture, heart disease, toe flexor strength, and foot morphology as the explanatory variables. The correlation coefficients of age, toe flexor strength, hallux valgus angle, arch height ratio, and spread ratio were all ≤0.7. Fracture, heart disease, and toe flexor strength were significant determinants of the ambulating ways.

**Table 2:** Factors affecting the ambulating ways identified in the multiple logistic regression analysis

	Partial regression coefficient	*p*-value	Odds ratio	95% confidence interval	
				Lower limit	Upper limit
Fracture	−1.802	0.006	0.165	0.046	0.589
Heart disease	− 1.478	0.032	0.228	0.059	0.884
Toe flexor strength	−0.320	0.000	0.726	0.610	0.865
Constant	3.974	0.000	53.177		

Model chi-square test, *p*<0.001

Percentage of correct classifications, 78.6%

Table 3 shows the proportional differences in muscle strength of the wheelchair group compared with the mean values of the walking group. As shown in Table 1, in the walking group, the mean (SD) hand grip and toe flexor strengths were 33.54 (9.38) and 10.01 (4.26), respectively. The average percentages of participants in the wheelchair group to these values of hand grip and toe flexor strength were 74.24% (22.42%) and 59.65% (31.45%), respectively. Toe flexor strength was significantly decreased compared to the hand grip strength (*p* = 0.004).

**Table 3:** Proportional differences in muscle strength in the wheelchair group compared with the mean values of the walking group

	Hand grip strength	Toe flexor strength	*p*-value
Proportional difference from walking mean, %	74.24 (22.42)	59.65 (31.45)	0.004

Hand grip strength and toe flexor strength are expressed as means (standard deviation)

## Discussion

This cross-sectional study examined the differences in toe flexor strength and foot morphology between older individuals who use a wheelchair and those who walk in everyday life. We found that older people who used a wheelchair were more likely to have a history of fracture or heart disease and weaker toe flexor strength. A wheelchair is often used when it is difficult to walk. The participants in this study did not have motor paralysis and were capable of standing with or without assistance, but they could not walk safely. A history of bone fracture or heart disease had a significant negative effect on the likelihood of walking. However, it is unclear whether the participants who used a wheelchair did so because of joint contracture subsequent to a fracture or disuse atrophy associated with heart disease, or whether the turning point for using a wheelchair was atrophy as a result of other diseases.

In this study, no significant between-group difference was found in foot morphology, including hallux valgus; even in the multiple logistic regression analysis, foot morphology was not among the significant factors. The prevalence of hallux valgus detected on radiographs in older Japanese men and women living in the community was 28.4% [4]. In this study, hallux valgus was found in approximately half of the older participants who needed some assistance with activities of daily living and were undergoing or had received ambulatory rehabilitation in a long-term care health facility. This percentage may be higher than that in community-dwelling older people.

Toe flexor strength was significantly greater in the walking group than in the wheelchair group. The strength of the toe flexors is related to walking parameters, such as the walking speed [11] and the timed up and go test [20]. In particular, a positive correlation between the toe flexor strength and the average number of steps taken per day was reported in women aged > 80 years [12]. Hallux valgus and lesser toe deformities were associated with reduced strength of plantar flexion [5, 21, 22]. In our study, no significant difference was found in foot morphology between the two groups; however, the toe flexor strength was significantly greater in the walking group, which was attributed to a difference in the amount of activities performed when standing, including walking.

Interestingly, the proportion of toe flexor strength was significantly lower than that of hand grip strength in the wheelchair group. With aging, the skeletal muscle mass in the lower limbs decreases to become significantly less than that in the upper limbs on magnetic resonance imaging [23]. The ratio of muscle thickness in older people to that in young people was significantly lower in the ankle plantar flexors than in the elbow flexors [24]. Similarly, there was a significant age-related decrease in the ratio of reduction in toe flexor strength to hand grip strength [25, 26]. The results of this study suggested that the standing and walking frequencies had some influence on these values because no difference was found in age between the walking and wheelchair groups.

A study has reported that the toe flexor muscles and ankle range of motion are important for balance while standing and maintaining functional ability [9]. Wheelchair users have significantly less toe flexor strength, which may affect balance when standing. Even when using a wheelchair as a way of ambulating in daily living, in order to stand, turn, and sit safely when transferring from a wheelchair, if there is no severe motor paralysis of the lower limbs, joint contracture, or load limitation, the person must be able to stand and may need to maintain toe flexor strength. The decrease in toe flexor strength in older adults who use wheelchairs without significant motor paralysis is thought to be attributed to disuse atrophy of the intrinsic and extrinsic muscles. Therefore, it may be recommended to perform toe flexion exercises while sitting.

This study had several limitations. First, as it was a cross-sectional study, the causal relationship between toe flexor strength and foot morphology remains unknown. Second, foot morphology was measured from the contour of the foot; therefore, the influence of edema subsequent to heart disease and the involvement of pronation or supination of the ankle joint due to loading on the sole of the foot while in the sitting position could not be determined. Third, those who were living in a facility or used its services had little information regarding previous hospitalizations and medical care at the facility. Therefore, the period of wheelchair use and the date of injury (i.e., fracture) were unknown. Accordingly, the effect of the duration of being in a wheelchair is not clear. Fourth, regarding the sample size, there was no problem that the number of people in the small group was 10 or more multiplied by the explanatory variable [27]. The explanatory variables selected with forward stepwise were three items, and the number of samples was the minimum required. However, the currently recommended sample size criteria for developing prediction models, notably the events per variable criterion ≥10 rule of thumb, are insufficient to warrant appropriate sample size decisions [28]. Pre-calculating the sample size from the effect size, power, etc., is viewed as more appropriate [28]. Future research may need to take appropriate consideration of the sample size in this manner. Finally, the proportion of male participants was lower than that of female participants and, therefore, the effect of sex differences was unknown.

## Conclusions

In this study, we found no difference in the foot morphology between those who used a wheelchair and those who did not, as a way of ambulating in daily life, but a difference in toe flexor strength was found. The reduction in the toe flexor strength in people using a wheelchair was more remarkable than the reduction in hand grip strength. Even when using a wheelchair in daily life, it is necessary to maintain the toe flexor strength to perform a safe standing motion.

## Data Availability

The anonymized data are available from the corresponding author upon reasonable request.
